# Gut microbial signatures and cardiac-microbiota axis in Yili horses with divergent exercise-induced cardiac remodeling

**DOI:** 10.3389/fmicb.2025.1689293

**Published:** 2025-12-03

**Authors:** Yike Bao, Tongliang Wang, Wusiman Adina, Runchen Yao, Hongzhong Chu, Xinkui Yao, Jun Meng, Jianwen Wang, Wanlu Ren, Yaqi Zeng

**Affiliations:** 1 College of Animal Science, Xinjiang Agricultural University, Ürümqi, China; 2 Xinjiang Key Laboratory of Equine Breeding and Exercise Physiology, Ürümqi, China; 3 Xinjiang Yili Kazakh Autonomous Prefecture Animal Husbandry Station, Ürümqi, China

**Keywords:** metagenomics, racehorse, conditioning training, microbiota, echocardiography

## Abstract

This study aimed to investigate how different training outcomes affect the gut microbiota composition in racehorses. Twenty-six Yili horses underwent a 9-month conditioning training regimen under uniform husbandry and management conditions. Post-training, the horses were divided into an excellence group (D. Y group) and a general group (D. P group) based on their athletic performance, with the top 10 performers constituting the D. Y group and the bottom 10 the D. P group. Cardiac morphology and function were quantitatively assessed via echocardiography, and metagenomic sequencing was performed on fresh fecal samples. Results indicated that there were no significant differences in gut microbiota and echocardiographic parameters between the two groups prior to training. However, significant differences were observed post-training (*p* < 0.05). At the genus level, *Parabacteroides*, *Bacteroides*, and *Prevotella* exhibited significantly greater abundance n the D. Y group. LEfSe analysis showed that Prevotella was markedly enriched in the D. Y group (LDA > 4). Functional profiling indicated that multiple metabolic pathways were significantly enriched in global and overview maps, with map00534 and map00190 being particularly enriched in the D. Y group (LDA > 2). Within CAZymes genes, eight were significantly enriched in the D. Y group, including four glycoside hydrolase genes, two carbohydrate esterase genes, and two carbohydrate-binding module genes. Echocardiography revealed significant differences in seven parameters between the groups, with the D. Y group exhibiting notably higher LV_MASS_I and LVM values (*p* < 0.01). dbRDA analysis demonstrated a significant association between LV_MASS_I and LVM and the gut microbiota profile (*p* < 0.01). These findings suggest that training-induced cardiac remodeling, particularly the increase in LV_MASS_I and LVM, is closely related to alterations in gut microbiota, with *Prevotella* enrichment potentially serving as a marker of favorable adaptation to the training regimen. The study provides robust evidence for understanding the interaction between aerobic training, gut microbiota, and cardiac characteristics in racehorses, and highlights potential directions for optimizing athletic performance and probiotic strategies in equine athletes.

## 1 Introduction

The Yili horse is a renowned Chinese breed known for its combination of strength and speed, strong adaptability, and disease resistance. It is the only riding horse breed recognized by the industry standards of China’s Ministry of Agriculture and Rural Affairs (Ouyang et al., 2023). The gut microbiota represents one of the most intricate and diverse microbial ecosystems in the body, comprising bacteria, fungi, viruses, and other microorganisms (Kuziel and Rakoff-Nahoum, 2022). Evidence indicates that these communities contribute not only to digestion and nutrient uptake but also to the regulation of immune function (Wu et al., 2023), modulation of inflammatory processes (Qiu et al., 2022), maintenance of metabolic balance (Eckel, 2021), and influence on mental health (Eckel, 2021). In herbivores, microbial activity is essential for fermenting plant fibers into volatile fatty acids, serving as a major energy source (Costa and Weese, 2018). In equines, such fermentation provides metabolic energy and engages in diverse biochemical pathways, exerting a substantial influence on overall health and physical performance. The equine intestinal tract hosts a broad spectrum of microbial taxa, including fungi, parasites, protozoa, archaea, viruses, and bacteria (Boucher et al., 2024; Vasco et al., 2020), each fulfilling distinct roles along different intestinal segments and showing high susceptibility to variations in diet, environmental conditions, and antibiotic administration. Microbial metabolites generated within the host exert significant effects on physiological health, pathological states, developmental processes, and even behavioral patterns (Tao et al., 2021; Wang et al., 2020; Tanase et al., 2020).

Variations in athletic performance among horses are associated with distinct gut microbiota compositions. While dietary patterns substantially influence gut microbiota, exercise training is also recognized as a major environmental determinant capable of altering microbial composition (Huang et al., 2024; Grosicki et al., 2023; Bycura et al., 2021). The structure and functional capacity of the gut microbiota can, to some extent, influence an individual’s drive and physical capacity for exercise (Agirman and Hsiao, 2022; Xia et al., 2024; Inglis, 2024), thereby linking microbial attributes to equine athletic performance. Evidence indicates that at least 36 microbial MAGs are more abundant in racehorses than in non-racehorses. These MAGs harbor the complete key genes for primary metabolic pathways involved in fiber fermentation, leading to the production of acetate and butyrate, thereby conferring a greater ability to generate short-chain fatty acids (SCFAs) and offering a potential metabolic advantage for enhancing exercise capacity (Li et al., 2023). Currently, research on the gut microbiota of athletic horses remains relatively scarce. In particular, the association between gut microbiota and different training effects has not been fully explored, and the link between exercise-induced cardiac remodeling and gut microbiota remains largely unexplored. Existing literature primarily focuses on the impact of microbiota on equine health and disease. Research remains limited on how exercise training shapes the gut microbiota and how these changes, in turn, influence equine athletic performance and adaptability. The present study investigated the impact of different training outcomes on the gut microbiota of racehorses using metagenomic sequencing, with emphasis on microbial differences between excellence and general groups, as well as training-induced alterations in cardiac structure and function, aiming to elucidate the dynamic modulation of the equine gut microbiota and to identify specific bacterial taxa and cardiac parameters closely associated with exercise adaptation.

## 2 Materials and methods

### 2.1 Experimental design and animals

The study was conducted at the state-owned Yili Stud Farm in the Xinjiang Uygur Autonomous Region. Twenty-six 18-month-old Yili horses, all in good health, maintained under uniform feeding and management without prior training, underwent a standardized nine-month training regimen (Wang et al., 2025). Experimental horses were housed under identical environmental conditions and managed according to standardized husbandry protocols. Each horse was fed 8 kg/d of dry hay and 4 kg/d of concentrate supplement. The feed formulation consisted of corn (17.28%), bran (5.26%), soybean meal (9.26%), calcium dihydrogen phosphate (1.15%), salt (0.63%), premix (0.31%), methionine (0.19%), and dry hay (65.92%), totaling 100%. The nutritional profile of this feed included dry matter (95.31%), crude protein (12.69%), crude fat (1.77%), neutral detergent fiber (42.23%), acid detergent fiber (37.98%), crude ash (8.16%), calcium (0.86%), phosphorus (0.41%), and digestible energy (9.03 MJ/kg). All horses included in the study were in good health, as confirmed by a veterinarian. None of the participants had a history of clinical disease, nor had they received any antibiotic or nonsteroidal anti-inflammatory drug treatment.

Following this conditioning program, a performance trial was held. Based on race outcomes, the top 10 horses were classified as the excellence group (D. Y group), the lowest-performing 10 as the general group (D. P group), while the remaining six were excluded from the analysis. Fecal samples were collected and echocardiograms were performed prior to training initiation, followed by a test race. After the trial concluded, fecal samples were collected again and echocardiograms were repeated, with metagenomic sequencing subsequently conducted.

### 2.2 Sample collection

After completing a 9-month training program, fecal samples were collected from the horses 3 days post-training. Horses were fasted for 12 h prior to sampling to minimize dietary intake’s impact on gut microbiota composition. All samples were collected on the morning of the fourth day to ensure consistency in environmental conditions and operational procedures. Samples were immediately stored in a −80 °C freezer until DNA extraction to prevent degradation and alteration of the microbial communities. Cardiac structural parameters (left ventricular internal diameter, diastolic left ventricular internal diameter, left ventricular posterior wall thickness, etc.) and functional indices (stroke volume, ejection fraction, cardiac index, etc.) were evaluated via echocardiography under resting conditions. Functional data were subsequently processed and analyzed using Excel spreadsheets. In this study, no sedatives were administered to any horses during echocardiographic examinations.

The 2D M-mode imaging was conducted using a Mindray M6 portable veterinary color Doppler ultrasound system operating at 2.5 MHz. Two image acquisitions were obtained from the right thoracic wall between the third and fourth or fourth and fifth intercostal spaces. The imaging depth was set to 30 cm, the transducer focus fixed at 5 cm, and the scanning angle maintained at 110°. All echocardiographic assessments were performed by a single experienced operator, and the mean value of two measurements per parameter was recorded. The protocol included right parasternal long-axis B-mode images at end-diastole and end-systole, right parasternal B-mode views of the left ventricular outflow tract, and right parasternal short-axis B/M-mode static and dynamic views. For consistency, three non-consecutive cardiac cycles were selected for analysis, with heart rates stabilized between 32 and 45 bpm, and mean values subsequently calculated. Transducer positioning was guided by intracardiac anatomical landmarks in accordance with established reference procedures. From the right parasternal short-axis view, a total of 31 cardiac dimensional parameters were measured.

During echocardiography, transducer positioning was guided by intracardiac anatomical landmarks in accordance with established protocols. From the right parasternal short-axis view, eight primary parameters were recorded: left ventricular internal dimension at end-diastole (LVIDd), left ventricular internal dimension at end-systole (LVIDs), right ventricular dimension at end-diastole (RVDd), right ventricular dimension at end-systole (RVDs), interventricular septal thickness (IVSd, IVSs), and left ventricular free wall thickness (LVFWd, LVFWs). In the right parasternal long-axis view, 15 parameters were assessed, including left ventricular volume (EDV, ESV), left ventricular minor dimension (LV Minor), mitral valve diameter (MVD), left atrial dimension (LADd, LADs), aortic root dimension (AODd, AODs) and additional structural indices. Derived calculations included the left atrium to aortic root (LA/AOD) ratio, pulmonary artery dimension (PAd, PAs), left ventricular mass parameters (LVW, LV MASS-I), left ventricular wall thickness parameters (MWTd, and RWTd), yielding a total of 23 morphological measurements. Functional assessment included ejection fraction (EF), left ventricular ejection time (LVET), stroke volume (SV), stroke volume index (SI), cardiac output (CO), cardiac index (CI), fractional shortening (FS), and velocity time integral (VTI), obtained through direct measurement and formula-based computation (Peng et al., 2024).

### 2.3 Metagenomic sequencing

Automated soil-fecal DNA extraction kit, Novazene (brand), DM401-C2 (catalog number). After DNA extraction from fecal samples, DNA concentration was precisely quantified using Qubit, and fragment integrity was assessed with the Qsep400 high-throughput bio-fragment analyzer. Qualified samples underwent enzymatic fragmentation to approximately 300 bp, followed by library preparation involving end-repair, A-tailing, adapter ligation, purification, and PCR amplification. Post-construction, preliminary quantification and insert size assessment were conducted with Qubit4.0. Libraries meeting quality criteria were pooled according to mass concentration and required target data output, then subjected to single-stranded circularization, followed by preparation of DNB nanospheres for DNA fragment sequencing on the DNBSEQ T7 platform.

### 2.4 Metagenomic data analysis

Raw sequencing data were processed with fastp to generate high-quality clean data for downstream analyses, removed reads containing low-quality bases (quality score < 15) exceeding a specified threshold (default: 40%); Removed reads with N bases exceeding a specified threshold (default: 5 bp); Removed adapter sequences (fastq default values); removed sequences shorter than 15 bp. For each sample, clean data were assembled using MEGAHIT with the following parameters: –k-list 21, 41, 61, 81, 91 –min-contig-len 500, and assembly metrics were assessed with QUAST. ORFs were predicted from assembled contigs (≥500 bp) using MetaGeneMark with default parameters for individual and pooled samples. Predicted ORFs from all datasets were merged, and redundant sequences were removed with CD-HIT. The resulting representative sequences were defined as unigenes, clustered at 95% sequence identity and 90% coverage, select the longest sequence as the representative sequence; use the following parameters: −c 0.95, −G 0, -aS 0.9, −g 1, −d 0. Clean data from each sample were aligned to the unigene set with Bowtie2 to finalize the unigene dataset for subsequent analyses, alignment parameters: -I 200, −X 400. Taxonomic assignments for unigenes were obtained through alignment against the MicroNR database, enabling classification at multiple taxonomic levels. Species-level annotation results were used to assess alpha and beta diversity. Functional annotations were derived by aligning unigenes to databases such as KEGG and CAZy.

### 2.5 Statistical analysis

All statistical analyses were performed using R software. Correlation heatmaps were generated with ComplexHeatmap 2.12.0; LEfSe analysis first employs the Kruskal-Wallis rank-sum test to identify species exhibiting significant abundance differences across groups. Subsequently, the Wilcoxon rank-sum test is used to assess the consistency of differences within subgroups. Finally, Linear Discriminant Analysis (LDA) is applied to estimate the magnitude of each species’ contribution to intergroup differences, yielding LDA scores. KEGG enrichment analysis was performed using R version 3.5.1 with the following packages and versions: ggplot2 3.3.0; dbRDA plots were generated using vegan 2.5.6 with a default VIF threshold of 10 (no value less than 0). This threshold was applied to exclude environmental factors exhibiting multicollinearity, where VIF values exceeding 10 are typically considered indicative of collinearity. Microbial abundance plots were generated using GraphPad Prism software (v9.4.1). Intergroup differences (e.g., cardiac ultrasound measurements, differentially expressed functional genes) underwent independent Student’s *t*-tests. Significance levels were defined as: ^ns^*P* > 0.05, *p < 0.05, **p < 0.01.

## 3 Results

### 3.1 Comprehensive analysis post-training

**Figure 1:**
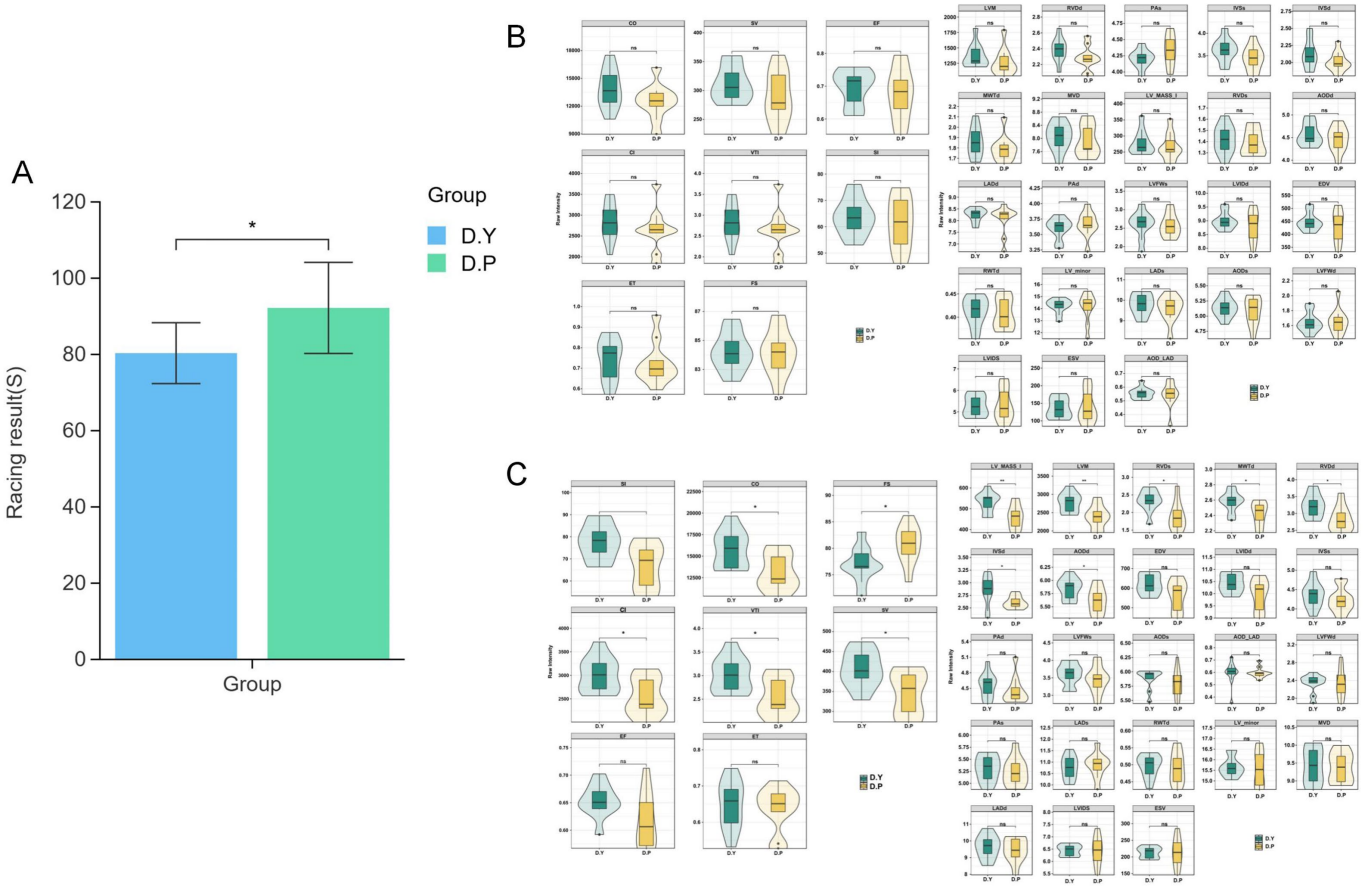
**(A)** Histogram of race performance differences; violin plot of cardiac dimensional indicators; **(B)** 31 cardiac functional and dimensional indices before training; **(C)** 31 cardiac functional and dimensional indices after training. Significance was determined using Student’s *t*-test: ^ns^*P* > 0.05, *p < 0.05, **p < 0.01.

In Figure 1A, race performance differed significantly between the two groups (*p* < 0.05). Evaluation of 31 cardiac dimensional indices revealed no pre-training differences in echocardiographic parameters (Figure 1B). Post-conditioning training analysis (Figure 1C) identified significant alterations in seven structural indices, with LVM and LV_MASS_I markedly higher in the D. Y group compared with the D. P group (*p <* 0.01), and RVDs, MWTd, IVSd, RVDd, and AODd also elevated in the D. Y group (*p <* 0.05). For functional parameters, SI, CI, VTI, SV, and CO were significantly greater in the D. Y group (*p <* 0.05). Collectively, the data indicate that conditioning training alone induces substantial remodeling of equine cardiac structure, accompanied by superior structural and functional cardiac profiles in the D. Y group relative to the D. P group. Detailed information on the structure and functional indicators of the horse heart is provided in Supplementary Table S1.

### 3.2 Metagenomic sequencing data information

Metagenomic sequencing of 20 equine fecal samples produced raw data that underwent adapter trimming and removal of low-quality reads, resulting in approximately 236 GB of high-quality sequences. Following per-sample quality control, the clean data were subjected to *de novo* assembly, and CDS prediction was subsequently conducted. Short contigs were excluded from further analysis. Gene identification prior to deduplication yielded 9,742,585 genes, whereas post-deduplication analysis identified 2,991,846 complete genes, representing 30.71%, the Q30 value was adopted as the assessment criterion for sequencing quality, where Q30 denotes the proportion of bases with an error rate below 1%. In this study, all samples achieved a Q30 value exceeding 95% of the total (Table 1).

**Table 1:** Statistical table of data preprocessing.

Sample	CleanData_bases/G	CleanData_Q20/%	CleanData_Q30/%	CleanData_GC/%
D.1	13.15	97.98	95.66	45.58
D.2	10.88	97.89	95.44	44.91
D.3	12.2	97.89	95.44	44.92
D.4	12.16	97.91	95.53	44.33
D.5	9.35	98.1	95.84	45.77
D.6	14.3	97.91	95.53	42.81
D.7	13.32	98.03	95.78	44.08
D.8	12.54	98	95.63	45.12
D.9	13.46	98.26	96.16	46.11
D.10	11.47	98.31	96.28	44.06
D.11	13.56	97.97	95.65	43.73
D.14	11.76	99.14	97.73	46.88
D.15	11.18	99.06	97.55	45.85
D.16	11.76	99	97.41	47.14
D.18	11.57	99.11	97.66	46.71
D.20	11.15	98.96	97.3	47.45
D.22	9.98	99.01	97.42	46.88
D.23	11	99.11	97.62	46.58
D.24	10.78	99.03	97.42	47.12
D.25	10.51	98.98	97.35	49.05

Sequencing statistics: Description: Sample: Indicates the sample name; Clean: Represents filtered valid data; bases: Number of bases in the data; Q20: Percentage of bases with a sequencing error rate below 0.01 (quality score > 20); Q30: Percentage of bases with a sequencing error rate below 0.001 (quality score > 30); GC: GC content of bases in the data.

### 3.3 Effects of different training regimens on gut microbiota

#### 3.3.1 Species composition of equine gut microbiota after training

Goods coverage values, which reflect sequencing depth, approached 1 in both groups, indicating adequate sequencing depth (>99.99%). Taxonomic classification was derived by aligning unigene protein sequences with the MicroNR database via DIAMOND, generating annotations across multiple taxonomic ranks. Relative abundance data at each level were used to identify the 10 most abundant taxa per group, with remaining taxa aggregated as “Others.”

**Figure 2:**
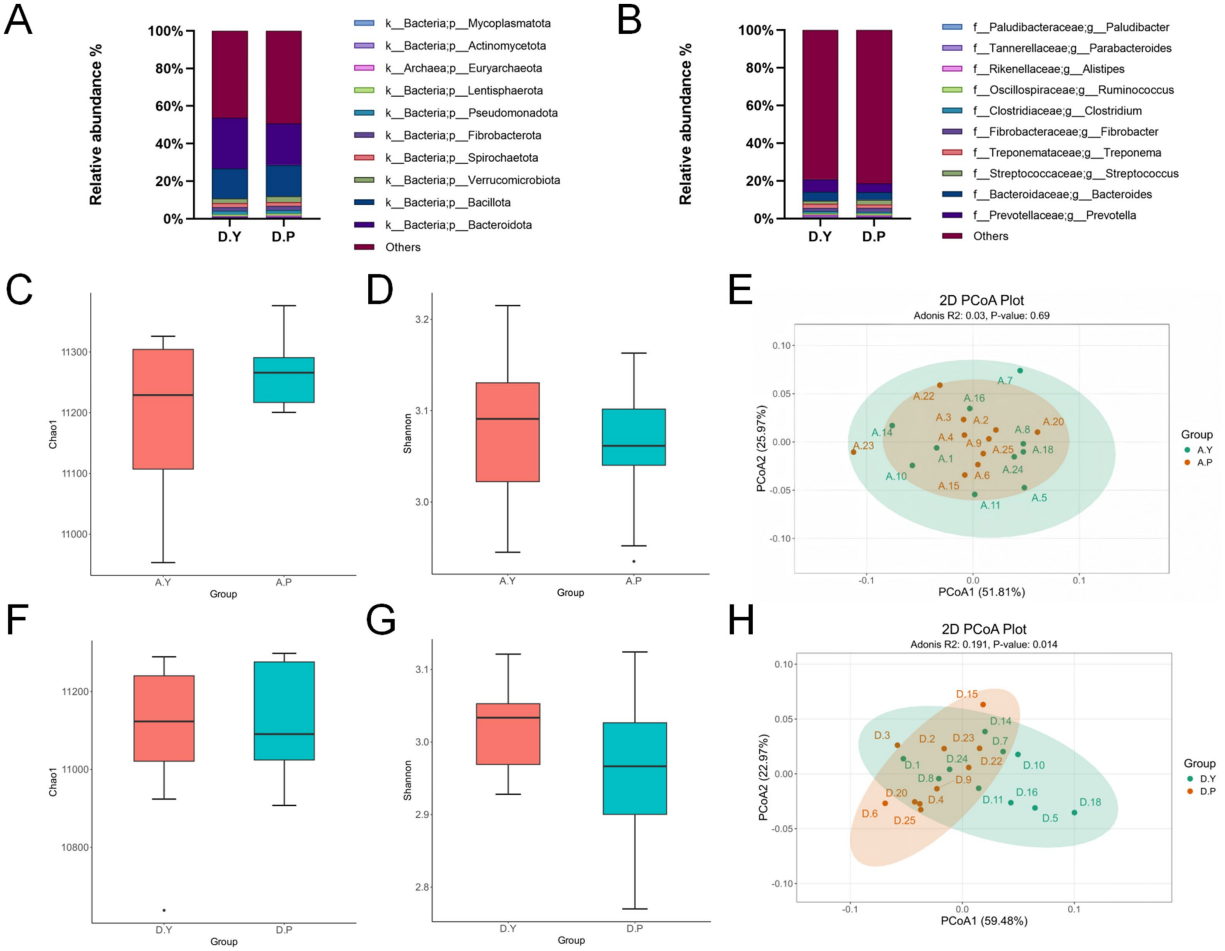
**(A)** Phylum-level species composition of equine gut microbiota. **(B)** Genus-level species composition of equine gut microbiota; pre-training phase: **(C)** comparison of equine gut microbiota α-diversity (Chao1 index) among groups. **(D)** Comparison of α-diversity (Shannon index) among groups. **(E)** Principal coordinate analysis (PCoA) based on phylum-level Bray–Curtis distance; post-training period. **(F)** Comparison of α diversity (Chao1 index) among groups. **(G)** Comparison of α diversity (Shannon index) among groups. **(H)** Principal coordinate analysis (PCoA) based on phylum-level Bray–Curtis distance.

At the phylum level (Figure 2A and Supplementary Table S2), the dominant taxa in both groups included *Bacteroidota*, *Bacillota*, *Verrucomicrobiota, Spirochaetota, Fibrobacterota, Pseudomonadota, Lentisphaerota, Euryarchaeota, Actinomycetota*, and *Mycoplasmatota*. *Bacteroidota* abundance was significantly higher in the D. Y group compared with the D. P group (*p* < 0.05).

At the genus level (Figure 2B and Supplementary Table S2), the principal taxa comprised *Prevotella*, *Bacteroides*, *Streptococcus, Treponema, Fibrobacter, Clostridium, Ruminococcus, Alistipes, Parabacteroides*, and *Paludibacter*. In the D. Y group, the abundance of *Bacteroides* and *Parabacteroides* species was significantly higher than in the D. P group (*p* < 0.05), and the abundance of *Prevotella* in the D. Y group was extremely significantly higher than in the D. P group (*p* < 0.01).

#### 3.3.2 Gut microbiota community structure

To investigate the effects of exercise training on equine gut microbiomes, we analyzed changes in gut microbial communities in two groups of horses during the pre-training phase (Phase A) and post-training phase (Phase D). α-diversity analysis employed the Chao 1 index to measure diversity and the Simpson index to assess evenness. Results indicated no significant differences in fecal microbial community structure or composition among all experimental horses prior to training initiation. After training, although no significant difference in α-diversity was observed between groups, gut microbial community structure analysis based on Bray-Curtis distance and principal coordinate analysis (PCoA) revealed distinct separation of data points from different groups along the PCoA1 and PCoA2 axes, indicating significant intergroup differences. Adonis analysis further validated these findings, yielding an *R*^2^ value of 0.191 and a *p*-value of 0.014 (*p* < 0.05), confirming significant differences in species diversity between the two groups of horses’ gut microbiomes (see Figures 2C–H).

#### 3.3.3 Differential species of gut microbiota in horses with distinct training outcomes

**Figure 3:**
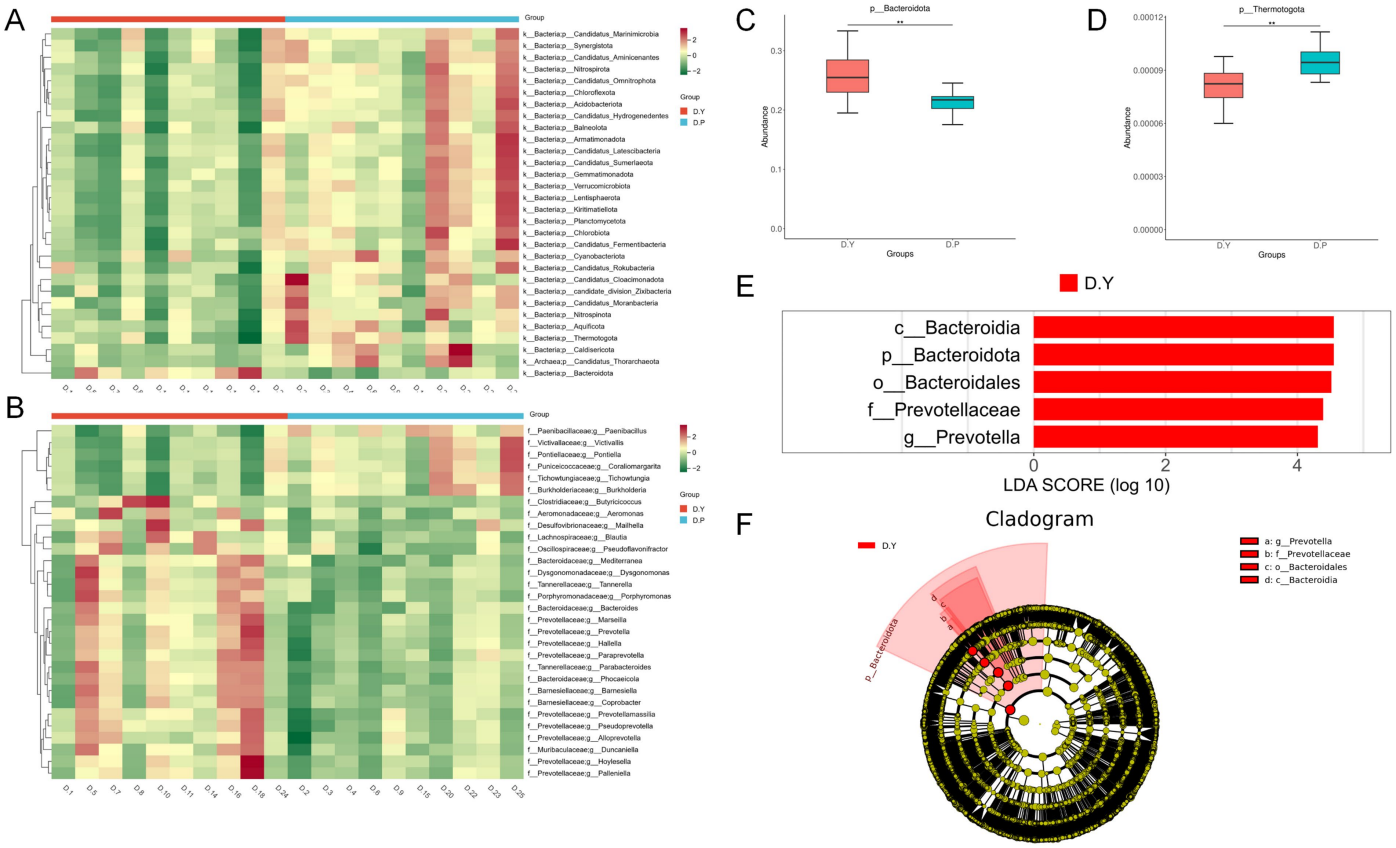
**(A)** Heatmap of species abundance differences at the door level. **(B)** Heatmap of species abundance differences at the genus level; Box plots of differentially abundant species: **(C)** Bacteroidetes, **(D)** thermotogota; **(E)** histogram of LDA score distributions; **(F)** phylogenetic tree based on LEfSe analysis.

Metastats analysis of species abundance across taxonomic levels produced heatmaps illustrating differences in microbial composition (Figures 3A,B). Significant variation was identified in 46 species at the phylum level and 200 species at the genus level, with the 30 most abundant genera presented. Notably, *Bacteroidota* exhibited significantly greater abundance in the D. Y group, whereas *Thermotogota* displayed markedly higher abundances in the D. P group (Figures 3C,D).

Further characterization of group-specific microbial signatures through LEfSe analysis and phylogenetic mapping (Figures 3E,F) identified Prevotella as the sole genus with a significantly elevated abundance in the D. Y group, with an LDA score exceeding 4. This enrichment suggests that Prevotella may serve as a representative taxonomic indicator associated with enhanced training outcomes. Detailed information on the differences in gut microbiota between the two groups is provided in Supplementary Table S3.

### 3.4 Effect of conditioning training on microbiome functional gene diversity

#### 3.4.1 KEGG analysis of gut microbiota metagenomic sequencing results

**Figure 4:**
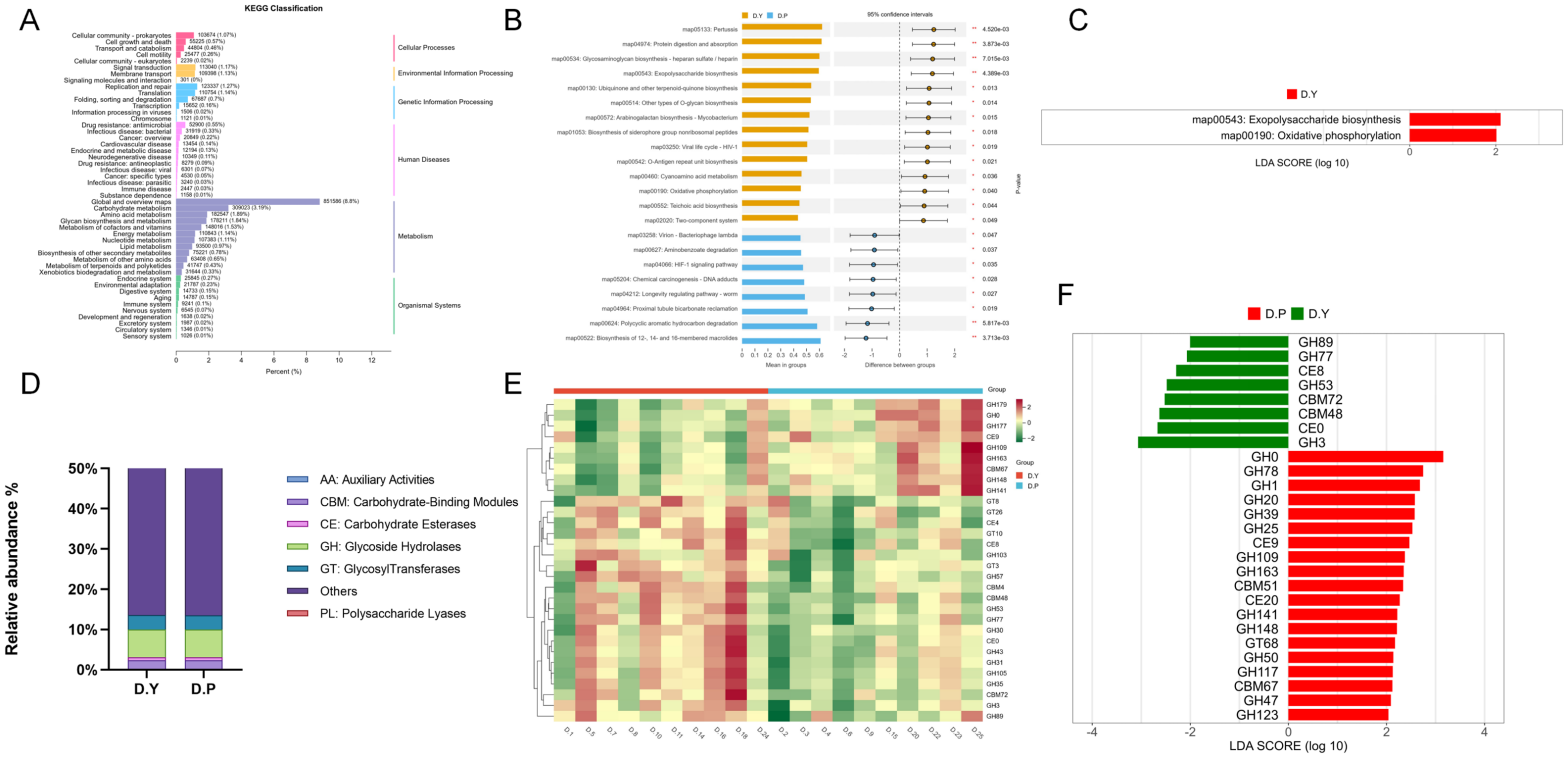
**(A)** KEGG metabolic pathway-related functional gene secondary level statistics. **(B)** Histogram of differentially functional genes. **(C)** Histogram of KEGG LDA value distribution. **(D)** Distribution of CAZy carbohydrate-active enzyme genes. **(E)** Heatmap of CAZy differential functional genes. **(F)** Histogram of CAZy LDA value distribution. All intergroup comparisons were performed using Student’s *t*-test. Significance symbols: ns indicates *p* > 0.05, *p < 0.05, **p < 0.01.

According to KEGG-based functional gene annotations, analysis at the level 2 pathway classification (Figure 4A) revealed that the most abundant functional gene categories in all groups were associated with global and overview maps, followed by metabolic processes such as carbohydrate metabolism and amino acid metabolism. Comparative analysis of level 3 metabolic pathways (Figure 4B) identified 25 pathways exhibiting significant inter-group differences (*p* < 0.05). Specifically, map00534: Glycosaminoglycan biosynthesis – heparan sulfate/heparin, map00543: Exopolysaccharide biosynthesis, and map04974: Protein digestion and absorption were markedly more prevalent in the D. Y group compared with the D. P group, whereas map00624: Polycyclic aromatic hydrocarbon degradation and map00522: Biosynthesis of 12-, 14-, and 16-membered macrolides were enriched in the D. P group. LEfSe analysis (LDA > 2) (Figure 4C) further indicated that map00534: Glycosaminoglycan biosynthesis – heparan sulfate/heparin and map00190: Oxidative phosphorylation were more enriched in the D. Y group (Supplementary Table S4).

#### 3.4.2 Comparative analysis of carbohydrate-active enzyme functions

Functional gene profiles were annotated using CAZy, with results presented in Figure 4D. At the class level, six categories of carbohydrate-active enzyme genes were identified, among which glycoside hydrolases constituted the largest proportion, followed by glycosyltransferases, carbohydrate-binding modules, carbohydrate esterases, polysaccharide lyases, and auxiliary activities. Heatmaps and LEfSe analysis (LDA > 2) indicated that within the CAZyme gene set, eight genes were enriched in the D. Y group, comprising four GH, two CE, and two CBM genes, whereas 19 genes were enriched in the D. P group, including 14 GH, one GT, two CBM, and two CE genes (Figures 4E,F and Supplementary Table S5).

#### 3.4.3 Cluster analysis of functional relative abundance

**Figure 5:**
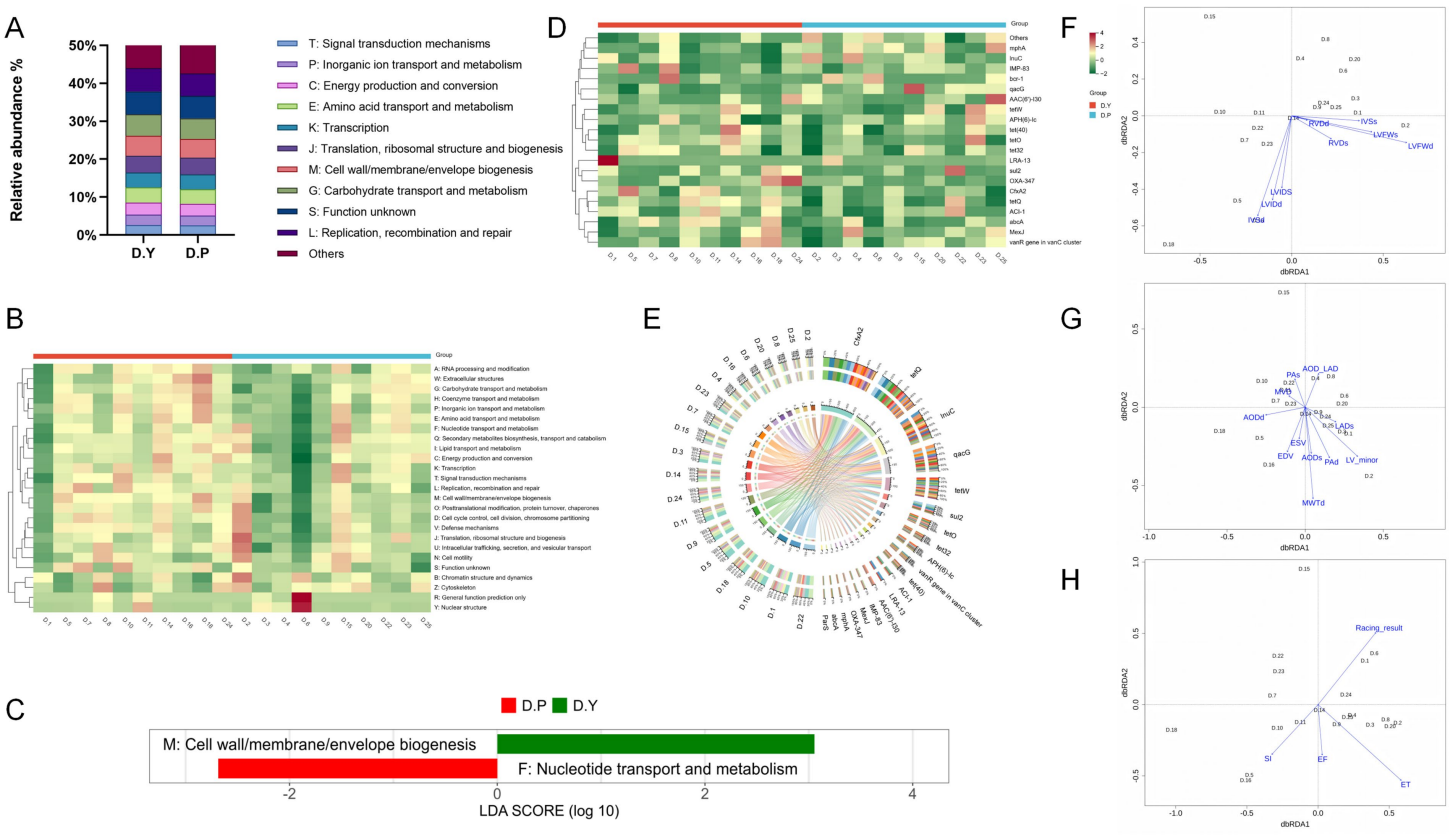
**(A)** Distribution of EggNOG gene composition. **(B)** Heatmap of EggNOG differential functional genes. **(C)** Histogram of EggNOG LDA value distribution. **(D)** CARD carbohydrate-active enzyme gene composition distribution. **(E)** CARD overview doughnut chart (see Supplementary Table S5 for details). The dbRDA Results Based on the Genus Level. **(F)** Eight key indices measured on the right parasternal short axis view; **(G)** 15 key indices obtained on the right parasternal long axis view; **(H)** eight cardiac function parameters and competition results. The default value for VIF is 10, and this value must not be less than 0. It is used to exclude environmental factors exhibiting multicollinearity, where a VIF value greater than 10 is typically considered indicative of collinearity.

Cluster analysis of relative abundance, performed using the EggNOG database, is presented in Figures 5A,B. The D. Y group exhibited the greatest abundance of L genes (Replication, Recombination, and Repair), whereas the D. P group contained the largest number of S genes (Unknown Function). LEfSe analysis (LDA > 2), illustrated in Figure 5C and Supplementary Table S5, revealed an enrichment of M genes (Cell Wall/Membrane/Envelope Biogenesis) in the D. Y group.

#### 3.4.4 Analysis based on antibiotic resistance genes

Genes were aligned against the CARD database to identify the 20 most abundant AROs. As illustrated in Figures 5D,E and Supplementary Table S5, the D. Y group exhibited a markedly higher relative abundance of the CfxA2 gene compared with the D. P group, whereas mphA and qacG displayed significantly greater relative abundance in the D. P group than in the D. Y group.

### 3.5 Correlation analysis between environmental factors and gut microbiota

As shown in Figures 5F–H, at the genus level, among the analyzed cardiac ultrasound indicators, LVFWd, MWTd, and ET showed significant correlations (*p* < 0.05) with equine gut microbiota, while LV_MASS_I and LVM exhibited extremely significant correlations (*p* < 0.01). This suggests they may play a crucial role in the relationship between equine cardiac health and gut microbiota structure. Among these, LV_MASS_I exhibited a high *R*^2^ value (*R*^2^ = 0.5219), indicating its strong explanatory power for gut microbial variation. The variance inflation factor (VIF) values for most cardiac ultrasound parameters were below 10, suggesting minimal multicollinearity issues in this study. This further confirms their stability within the model, demonstrating minimal susceptibility to multicollinearity effects. Specific details are provided in Supplementary Table S6.

## 4 Discussion

### 4.1 Correlation between different training outcomes and gut microbiota composition

Horses exhibiting different athletic performances display distinct gut microbiota profiles. Previous research has demonstrated that regular, appropriately structured exercise modifies gut microbial composition and exerts beneficial physiological effects (Yang et al., 2025; Czerwińska-Ledwig et al., 2024). These changes are primarily reflected in the composition of the gut microbiota, specifically in the significant differences in beta diversity observed between horses with varying athletic performance. Training has been linked to an increased abundance of beneficial intestinal taxa and enhanced overall microbial diversity, exercise can alter the composition of athletes’ gut microbiota, SCFA concentrations, and the prevalence of SCFA-producing microorganisms in athletes (Santos et al., 2023). Castro et al. (2021) reported similar exercise-induced alterations in gut microbiota diversity in mice. In the present study, conditioning training induced shifts in both taxonomic and functional gene diversity in equine gut microbiota. Greater inter-individual variation and higher diversity indices were observed in the D. Y group compared with the D. P group. PCoA analysis further demonstrated that post-training microbial community structures differed significantly between groups.

We found that the *R*^2^ value of the model before training was only 0.03, while after training it increased to 0.19. Although the *R*^2^ value improved post-training, it remained relatively low, potentially due to the consistency of the rearing environment. Since all horses were kept in the same rearing environment before and after training, the impact of environmental factors on gut microbial community structure may have been relatively stable, thereby reducing the model’s ability to explain variability attributable to environmental changes. Before training, as the horses had not yet undergone the training regimen, their gut microbiomes may have been more susceptible to the influence of the rearing environment. These effects might not have been fully captured by the model, leading to the low *R*^2^ value. After training, although the rearing environment remained unchanged, the training itself may have exerted an impact on the horses’ gut microbiomes. However, even when training introduced new influencing factors, the *R*^2^ value remained low. This suggests that other factors not captured by the model—such as genetic factors and individual variations—may still be influencing the gut microbial community structure.

### 4.2 Comparative analysis of gut microbiota compositions between groups

Exercise exerts a substantial influence on gut microbiota composition. At the phylum level, both *Bacteroidota* and *Bacillota* displayed high abundance. Previous research, including work by Motiani et al. (2020), has demonstrated that training modalities such as sprint interval training (SIT) and moderate-intensity continuous training (MICT) can modulate gut microbiota composition, often increasing the proportion of *Bacteroidota*. The present results align closely with those observations. *Bacteroidota*, a major constituent of the human gut microbiota, exhibits strong associations with a healthy microbiome (Tanca et al., 2024). In the current analysis, *Bacteroidota* abundance in the D. Y group was markedly greater than in the D. P group, potentially reflecting the effect of sustained, structured training in promoting its proliferation. In high-performing racehorses, SCFAs generated by *Bacteroidota* may enhance glucose synthesis efficiency under comparable training loads, reduce muscle glycogen utilization, and limit lactic acid accumulation, thereby creating a metabolic feedback loop that supports the continued dominance of *Bacteroidota*. *Bacillota* contributes to SCFA production through dietary fiber fermentation, which benefits endurance performance (Solouki et al., 2024). The equilibrium between *Bacillota* (*Firmicutes*) and *Bacteroidota* represents a central determinant in exercise-related health outcomes. An elevated *Firmicutes*/*Bacteroidota* (F/B) ratio is often associated with adverse metabolic effects (Yu et al., 2019; Hassan et al., 2024), whereas regular exercise—particularly aerobic training—tends to lower this ratio, typically through increased *Bacteroidota* abundance coupled with moderate reductions in *Bacillota* (Motiani et al., 2020). In the present study, we observed that the relative abundance of Bacteroidota was significantly higher in the D. Y group compared to the D. P group, while the abundance of Firmicutes was relatively lower. This resulted in a lower Firmicutes-to-Bacteroidota (F/B) ratio of 0.59 in the D. Y group, compared to 0.77 in the D. P group. This finding is in line with existing literature, which suggests that a lower F/B ratio is generally associated with a healthier metabolic status. The microbiota profile observed in the D. Y group may reflect an adaptive response to regular exercise training, which could potentially contribute to enhanced athletic performance and overall health in these animals. At the genus level, the D. Y group showed significantly elevated levels of *Parabacteroides*, *Bacteroides*, and *Prevotella*. *Parabacteroides* has been recognized as a potential next-generation probiotic. In a study by Yang et al. (2019) comparing gut microbiota in athletes and sedentary individuals, *Parabacteroides* was more prevalent in athletes and displayed a positive association with average weekly exercise duration. SCFAs, such as succinate, produced by *Parabacteroides* can enhance skeletal muscle glucose and lipid metabolism through activation of the AMPK pathway, acting synergistically with exercise to improve metabolic function (Frampton et al., 2020). *Bacteroides* and *Prevotella*, two core bacterial genera in the gut microbiota, exhibit abundances closely aligned with their functional roles (Mańkowska et al., 2022). According to Morita et al. (2023), exercise-induced acceleration of intestinal motility may increase the availability of mucus-derived polysaccharides as carbon sources, thereby elevating SCFA concentrations and the hepatic expression of gluconeogenesis-related enzymes while reducing hepatic glycogen stores; greater *Bacteroides* abundance is associated with enhanced endurance performance. *Prevotella*, characterized by strong carbohydrate fermentation capacity, generates SCFAs such as propionate (Betancur-Murillo et al., 2022). Li et al. (2023) found that the Prevotella abundance in elite racehorses was significantly higher than in non-racehorses (*p* < 0.05), suggesting an association between this genus and athletic ability. Its enrichment in racehorses may contribute to enhanced athletic performance. In this study, the *Prevotella* abundance in Group D. Y was significantly higher than in Group D. P (*p* < 0.05). Its enrichment in Group D. Y may be related to the group’s locomotor capacity and training status, further supporting the role of Prevotella in athletic performance. Y group showed significantly higher *Prevotella* abundance than the D. P group (*p* < 0.05). The enrichment in the D. Y group may be related to its athletic ability and training status, further supporting the potential association between Prevotella and athletic performance.

### 4.3 Potential association between differential microbial communities and enhanced athletic performance

The Metastats analysis revealed that Thermotogota, which was enriched in the D. P group, exhibits efficient carbohydrate catabolism and fermentation capabilities. This results in the production of metabolites such as hydrogen (H₂) and acetic acid (Ma et al., 2022). Excessive dependence on fiber degradation pathways, however, may result in surplus H₂ accumulation, disrupting intestinal redox equilibrium and reducing energy conversion efficiency, thereby impairing sprint capacity (Meng et al., 2022). To further identify taxa with statistically significant variation across training outcomes, LEfSe analysis based on LDA effect size was applied. *Prevotella* emerged as more abundant in the D. Y group. Previous research by DeVadder demonstrated that *Prevotella* enhanced host gluconeogenesis via propionic acid-mediated modulation of the gut–liver axis in murine models (De Vadder et al., 2016). In equines, specific *Prevotella* strains contribute to immune regulation through the production of butyrate and other anti-inflammatory metabolites (Dou et al., 2023). Such modulation mitigates post-exercise inflammation, accelerates recovery, and supports improved athletic output (Nieman et al., 2025).

Metabolic pathway annotation was performed to assess the influence of conditioning training on the functional gene diversity of racehorse microbiota under varying training outcomes. Comparative analyses with the CAZyme, KEGG, and eggNOG databases identified Global and Overview maps as the pathways with the highest relative abundance of functional genes across all groups, reflecting the integral role of microorganisms in systemic metabolism and functional regulation. Existing research indicates that metabolic pathway map00534 participates in tissue repair, angiogenesis, and anti-inflammatory responses by regulating multiple biological processes, including glycosaminoglycan metabolism (Demeter et al., 2024). In our study, the map00534 pathway showed significant enrichment in the D. Y group, potentially linked to this group’s pronounced advantage in the acetyl heparan sulfate synthesis and bacterial extracellular polysaccharide synthesis pathways. These pathways may collectively promote superior athletic performance by protecting the musculoskeletal system (e.g., joints) and maintaining homeostasis of the internal environment. Additionally, our analysis indicates that carbohydrate metabolism and amino acid metabolism also exhibit high activity. Carbohydrate metabolism appears to be a major energy-supplying route in high-performance racehorses, enabling rapid carbohydrate breakdown during intense exertion and ensuring adequate energy delivery for muscle contraction and physical performance (Myćka et al., 2023; Newell et al., 2018). Enhanced amino acid metabolism supports efficient energy conversion, improving nutrient utilization and maintaining energy equilibrium during exercise (Li and Hoppe, 2023). These findings suggest that these metabolic pathways may play a significant role in providing energy for athletic horses. Nonetheless, it is important to acknowledge that the mere presence of these pathways does not guarantee their active involvement in the metabolic processes. Consequently, the proposed link between these pathways and the athletic prowess of horses should be subjected to further experimental scrutiny to validate this hypothesis. In the D. Y group, the significant enrichment of metabolic pathway map00190 was attributed to the key domain of energy metabolism. Oxidative phosphorylation, as the core mechanism for ATP production within cells, enables more efficient utilization of oxygen and various substrates—such as fatty acids and glucose—to generate energy (Zhao and Gao, 2024). Houzelle et al. (2020) investigated the relationship between mitochondrial dynamics and oxidative capacity in human skeletal muscle, categorizing 45 participants into four distinct groups, including a control group and endurance athletes. The study revealed that endurance-trained athletes exhibited the highest levels of mitochondrial density markers. Mitochondrial dynamics were closely associated with oxidative capacity in human skeletal muscle and may play a crucial role in maintaining muscle insulin sensitivity. In our experiments, the map00190 pathway exhibited enrichment in the D. Y group, potentially linked to superior mitochondrial function in these horses, supporting their exceptional performance during high-intensity exercise.

Adjustments in microbial metabolic functions may align with host energy requirements and adaptations to training stimuli. Functional gene comparison with the CAZy database revealed a predominance of glycoside hydrolases, which likely serve as key catalysts in carbohydrate degradation and metabolism, highlighting carbohydrates as a primary energy substrate for the microbiome (Chandel, 2021). Hu et al. (2024) assembled 4,142 metagenomic genomes from equine gut microbiota and identified that the MAG117.bin13 genome contained the highest number of polysaccharide utilization loci (PULs). This indicates its high carbohydrate degradation capacity, with the GH family capable of breaking down complex polysaccharides (cellulose, hemicellulose, etc.) in forage to produce short-chain fatty acids (SCFAs) such as acetate and butyrate. Training may alter the microbiota’s carbohydrate utilization capacity. However, GH89, GH77, GH53, and GH3 have been relatively understudied in humans or animals, and even more scarce in horses. This may stem from differences in expression patterns and biological functions across species, coupled with a lack of in-depth research specifically targeting horses. Future studies should explore the functions and regulatory mechanisms of these enzymes in horses to better understand their roles in equine health and disease. Training can alter the microbiome’s capacity for carbohydrate utilization. However, GH89, GH77, GH53, and GH3 have been relatively understudied in humans and animals, with research in horses being even scarcer. This scarcity may stem from differences in the expression patterns and biological functions of these enzymes across species, coupled with a lack of in-depth studies specifically targeting horses. Future research should therefore focus on exploring the functions and regulatory mechanisms of these enzymes in horses to better understand their roles in equine health and disease.

In our EggNOG analysis, the D. Y group showed a notable abundance of genes in category L. This observation could imply that cells of athletic horses may be subjected to significant physiological stress during training. The cells in the superior group might possess enhanced DNA repair capabilities, potentially contributing to more effective maintenance of genomic stability. During training, tissues such as muscles undergo continuous adaptation and growth, where cell proliferation plays a critical role. DNA replication, recombination, and repair are vital steps in this process. A sufficient number of related genes could ensure smooth cell division and proliferation, providing ample cellular resources for tissue repair and growth (Witkowska-Piłaszewicz et al., 2020), which may support muscle development and overall performance improvement in athletic horses. Antibiotic resistance gene analysis further demonstrated elevated CfxA2 gene abundance in the D. Y group. CfxA2 encodes a β-lactamase capable of hydrolyzing β-lactam antibiotics (e.g., penicillin and cephalosporin), conferring bacterial resistance to these agents (Binta and Patel, 2016). Yokoyama et al. (2023) demonstrated that β-lactamase production in *Prevotella* was closely associated with the expression of cfxA and cfxA2 genes, aligning with the elevated *Prevotella* abundance observed in the D. Y group of this study. The substantial presence of the cfxA2 gene suggests that the gut microbiota of elite racehorses may possess heightened antibiotic resistance, potentially influencing overall health status. This enrichment could stem from a combination of factors, including immune modulation, nutrient metabolism, and adaptation to environmental conditions. Elucidation of the precise mechanistic pathways and the consequent health implications warrants further investigation.

### 4.4 Evaluation of the explanatory power of cardiac structure and function indicators for gut microbiota variation

Based on dbRDA analysis, significant associations were found between LVFWd, MWTd, and ET with gut microbiota (*p* < 0.05), while LV_MASS_I showed a highly significant association with gut microbial communities (*p* < 0.01). Notably, LV_MASS_I exhibited a high *R*^2^ value of 0.5219 and a VIF value of only 0.002, suggesting its potential as a biomarker for evaluating the structure and function of equine gut microbial communities. Cardiac remodeling may represent a physiological adaptation in athletes resulting from prolonged training (Ochten et al., 2025). LV_MASS_I and LVM could serve as important parameters for evaluating left ventricular hypertrophy. Evidence suggests that sustained training might induce myocardial hypertrophy, potentially promoting the development of the myocardial intercalated disk and myoblasts, thereby possibly increasing left ventricular mass and mass index (Wu et al., 2024). In well-trained racehorses, elevated oxygen and energy demands may require more efficient cardiac output, potentially enhancing left ventricular contractility and further increasing the mass index (Pınar and Sancak, 2018). The present study aligns with this pattern, as the D. Y group exhibited a significantly higher left ventricular mass index compared with the D. P group. In athlete cardiac remodeling research, MWTd is also recognized as a key metric (Christou et al., 2020). The elevated MWTd observed in the D. Y group might indicate structural adaptation of the myocardium to high-intensity training, which could enable more efficient blood ejection and better preservation of cardiac output during exertion (Koenig et al., 2017).

Regular physical training may lead to myocardial remodeling, accompanied by increases in LV_MASS_I and LVM, which could enhance stroke volume and aerobic capacity while potentially modulating gut microbiota composition. This modulation might favor the proliferation of taxa with dietary fiber-fermenting capabilities, such as *Prevotella*, allowing the utilization of fermentation-derived propionate as a gluconeogenic substrate to stabilize blood glucose and limit muscle glycogen depletion. Evidence suggests that either exogenous propionate supplementation or microbiota-mediated production could markedly extend endurance duration, enhance power output, and reduce biomarkers of exercise-induced fatigue (Carmody and Baggish, 2019)^.^ Propionate may further activate AMPK in skeletal muscle and cardiomyocytes, stimulate fatty acid oxidation, delay fatigue onset, limit LPS translocation, attenuate post-exercise inflammatory responses, preserve intestinal barrier function, and protect cardiomyocytes and myofibers against oxidative stress, thereby potentially sustaining the physiological myocardial remodeling induced by exercise (Liu et al., 2024).

### 4.5 Limitations analysis

This study systematically elucidates the differential effects of training on cardiac remodeling and gut microbiota in Ili horses. Although the sample size of 20 horses is sufficient for preliminary exploratory analysis, it may be inadequate to detect all potential biological differences. Future studies should consider increasing sample size to enhance statistical power and improve the generalizability of results. Host genetic factors and environmental heterogeneity may influence gut microbiota composition and function, thereby altering observed associations. Functional predictions based on metagenomic data inherently carry uncertainty due to the complexity of microbial interactions and limitations of current bioinformatics tools, necessitating experimental validation to confirm accuracy and relevance. Currently, this study collected the most comprehensive set of 31 cardiac indicators available, aiming for preliminary exploratory research into their changes during exercise-induced cardiac remodeling. Therefore, no multiple testing correction was applied. Future studies may identify independent predictors among relevant cardiac variables through variable selection and multivariate regression analysis. Although Prevotella and LV_MASS_I show promise as biomarkers, their specific roles in training adaptation and gut microbial function require further validation. Targeted metabolomics techniques (e.g., measuring short-chain fatty acids in fecal samples) could provide additional insights for metabolic prediction and help validate the functional significance of identified microbial markers, thereby addressing limitations of this study.

## 5 Conclusion

Metagenomic sequencing demonstrated that gut microbiota composition and functional profiles varied according to training outcomes, with *Parabacteroides*, *Bacteroides*, and *Prevotella* exhibiting significantly higher abundance in the D. Y group compared with the D. P group. Variations in the prevalence of specific taxa may reflect differences in training efficacy. Exercise training concurrently modulated gut microbiota characteristics and cardiac remodeling, thereby enhancing exercise adaptability. *Prevotella* could emerge as a representative biomarker of training outcome, while LV_MASS_I and LVM showed strong associations with microbial profiles. The proposed “Cardiac-microbiota axis” offers an explanatory framework for individual variability in performance and supports the formulation of evidence-based training strategies and dietary interventions.

## Data Availability

The datasets presented in this study can be found in online repositories. The names of the repository/repositories and accession number(s) can be found in the article/Supplementary material.

## References

[ref1] Agirman G. Hsiao E. Y. (2022). Gut microbes shape athletic motivation. Nature 612, 633–634. doi: 10.1038/d41586-022-04355-3, PMID: 36517676

[ref2] Betancur-Murillo C. L. Aguilar-Marín S. B. Jovel J. (2022). *Prevotella*: a key player in ruminal metabolism. Microorganisms 11:1. doi: 10.3390/microorganisms11010001, PMID: 36677293 PMC9866204

[ref3] Binta B. Patel M. (2016). Detection of cfxA2, cfxA3, and cfxA6 genes in beta-lactamase producing oral anaerobes. J. Appl. Oral Sci. 24, 142–147. doi: 10.1590/1678-775720150469, PMID: 27119762 PMC4836921

[ref4] Boucher L. Leduc L. Leclère M. Costa M. C. (2024). Current understanding of equine gut dysbiosis and microbiota manipulation techniques: comparison with current knowledge in other species. Animals 14:758. doi: 10.3390/ani1405075 38473143 PMC10931082

[ref5] Bycura D. Santos A. C. Shiffer A. Kyman S. Winfree K. Sutliffe J. . (2021). Impact of different exercise modalities on the human gut microbiome. Sports 9:14. doi: 10.3390/sports9020014, PMID: 33494210 PMC7909775

[ref6] Carmody R. N. Baggish A. L. (2019). Working out the bugs: microbial modulation of athletic performance. Nat. Metab. 1, 658–659. doi: 10.1038/s42255-019-0092-1, PMID: 32694643

[ref7] Castro A. P. Silva K. K. S. Medeiros C. S. A. Alves F. Araujo R. C. Almeida J. A. (2021). Effects of 12 weeks of resistance training on rat gut microbiota composition. J. Exp. Biol. 224:2543. doi: 10.1242/jeb.242543, PMID: 34137868

[ref8] Chandel N. S. (2021). Carbohydrate metabolism. Cold Spring Harb. Perspect. Biol. 13:a040568. doi: 10.1101/cshperspect.a040568, PMID: 33397651 PMC7778149

[ref9] Christou G. A. Pagourelias E. D. Anifanti M. A. Sotiriou P. G. Koutlianos N. A. Tsironi M. P. . (2020). Exploring the determinants of the cardiac changes after ultra-long duration exercise: the echocardiographic Spartathlon study. Eur. J. Prev. Cardiol. 27, 1467–1477. doi: 10.1177/2047487319898782, PMID: 32013601

[ref10] Costa M. C. Weese J. S. (2018). Understanding the intestinal microbiome in health and disease. Vet. Clin. N. Am. Equine Pract. 34, 1–12. doi: 10.1016/j.cveq.2017.11.005, PMID: 29402480

[ref11] Czerwińska-Ledwig O. Nowak-Zaleska A. Żychowska M. Meyza K. Pałka T. Dzidek A. . (2024). The positive effects of training and time-restricted eating in gut microbiota biodiversity in patients with multiple myeloma. Nutrients 17:61. doi: 10.3390/nu17010061, PMID: 39796496 PMC11722647

[ref12] De Vadder F. Kovatcheva-Datchary P. Zitoun C. Duchampt A. Bäckhed F. Mithieux G. . (2016). Microbiota-Produced Succinate Improves Glucose Homeostasis via Intestinal Gluconeogenesis. Cell Metab. 24, 151–157. doi: 10.1016/j.cmet.2016.06.013, PMID: 27411015

[ref13] Demeter F. Peleskei Z. Kútvölgyi K. Rusznyák Á. Fenyvesi F. Kajtár R. . (2024). Synthesis and biological profiling of seven heparin and Heparan Sulphate analogue Trisaccharides. Biomolecules 14:1052. doi: 10.3390/biom14091052, PMID: 39334821 PMC11429564

[ref14] Dou L. Liu C. Chen X. Yang Z. Hu G. Zhang M. . (2023). Supplemental *Clostridium butyricum* modulates skeletal muscle development and meat quality by shaping the gut microbiota of lambs. Meat Sci. 204:109235. doi: 10.1016/j.meatsci.2023.109235, PMID: 37301103

[ref15] Eckel J. (2021). Intestinal microbiota and host metabolism — a complex relationship. Acta Physiol. 232:3638. doi: 10.1111/apha.13638, PMID: 33638283

[ref16] Frampton J. Murphy K. G. Frost G. Chambers E. S. (2020). Short-chain fatty acids as potential regulators of skeletal muscle metabolism and function. Nat. Metab. 2, 840–848. doi: 10.1038/s42255-020-0188-7, PMID: 32694821

[ref17] Grosicki G. J. Langan S. P. Bagley J. R. Galpin A. J. Garner D. Hampton-Marcell J. T. . (2023). Gut check: unveiling the influence of acute exercise on the gut microbiota. Exp. Physiol. 108, 1466–1480. doi: 10.1113/EP091446, PMID: 37702557 PMC10988526

[ref18] Hassan N. E. El-Masry S. A. Shebini S. M. E. Ahmed N. H. Mohamed T. F. Mostafa M. I. . (2024). Gut dysbiosis is linked to metabolic syndrome in obese Egyptian women: potential treatment by probiotics and high fiber diets regimen. Sci. Rep. 14:5464. doi: 10.1038/s41598-024-54285-5 38443406 PMC10914807

[ref19] Houzelle A. Jörgensen J. A. Schaart G. Daemen S. van Polanen N. Fealy C. E. . (2020). Human skeletal muscle mitochondrial dynamics in relation to oxidative capacity and insulin sensitivity. Diabetologia 64, 424–436. doi: 10.1007/s00125-020-05335-w, PMID: 33258025 PMC7801361

[ref20] Hu L. Li X. Li C. Wang L. Han L. Ni W. . (2024). Characterization of a novel multifunctional glycoside hydrolase family in the metagenome-assembled genomes of horse gut. Gene 927:148758. doi: 10.1016/j.gene.2024.148758, PMID: 38977109

[ref21] Huang B. Zhao L. Campbell S. C. (2024). Bidirectional link between exercise and the gut microbiota. Exerc. Sport Sci. Rev. 52, 132–144. doi: 10.1249/JES.0000000000000343, PMID: 39190614

[ref22] Inglis G. A. S. (2024). Gut microorganisms enhance bone mass after exercise. Nat Aging 4, 905–905. doi: 10.1038/s43587-024-00676-2, PMID: 38987648

[ref23] Koenig T. R. Mitchell K. J. Schwarzwald C. C. (2017). Echocardiographic assessment of left ventricular function in healthy horses and in horses with heart disease using pulsed-wave tissue Doppler imaging. J. Vet. Intern. Med. 31, 556–567. doi: 10.1111/jvim.14641, PMID: 28109132 PMC5354014

[ref24] Kuziel G. A. Rakoff-Nahoum S. (2022). The gut microbiome. Curr. Biol. 32, R257–R264. doi: 10.1016/j.cub.2022.02.023, PMID: 35349808

[ref25] Li Q. Hoppe T. (2023). Role of amino acid metabolism in mitochondrial homeostasis. Front. Cell Dev. Biol. 11:1127618. doi: 10.3389/fcell.2023.1127618, PMID: 36923249 PMC10008872

[ref26] Li C. Li X. Guo R. Ni W. Liu K. Liu Z. . (2023). Expanded catalogue of metagenome-assembled genomes reveals resistome characteristics and athletic performance-associated microbes in horse. Microbiome 11:7. 36631912 10.1186/s40168-022-01448-z PMC9835274

[ref27] Liu C. Wong P. Y. Wang Q. Wong H. Y. Huang T. Cui C. . (2024). Short-chain fatty acids enhance muscle mass and function through the activation of mTOR signalling pathways in sarcopenic mice. J. Cachexia. Sarcopenia Muscle 15, 2387–2401. doi: 10.1002/jcsm.13573, PMID: 39482890 PMC11634463

[ref28] Ma S. Peng C. Deng Y. Zhang H. Yang Y.. (2022). Recent developments in phylum *Thermotoga*. China Biogas 40, 3–17. doi: 10.20022/j.cnki.1000-1166.2022050003

[ref29] Mańkowska K. Marchelek-Myśliwiec M. Kochan P. Kosik-Bogacka D. Konopka T. Grygorcewicz B. . (2022). Microbiota in sports. Arch. Microbiol. 204:485. doi: 10.1007/s00203-022-03111-5, PMID: 35834007 PMC9283338

[ref30] Meng Q. Zhang Y. Li J. Shi B. Ma Q. Shan A. (2022). Lycopene affects intestinal barrier function and the gut microbiota in weaned piglets via antioxidant signaling regulation. J. Nutr. 152, 2396–2408. doi: 10.1093/jn/nxac208, PMID: 36774106

[ref31] Morita H. Kano C. Ishii C. Kagata N. Ishikawa T. Hirayama A. . (2023). *Bacteroides uniformis* and its preferred substrate, α-cyclodextrin, enhance endurance exercise performance in mice and human males. Sci. Adv. 9:2120. doi: 10.1126/sciadv.add2120, PMID: 36696509 PMC9876546

[ref32] Motiani K. K. Collado M. C. Eskelinen J. J. Virtanen K. A. Löyttyniemi E. Salminen S. . (2020). Exercise training modulates gut microbiota profile and improves Endotoxemia. Med. Sci. Sports Exerc. 52, 94–104. doi: 10.1249/MSS.0000000000002112, PMID: 31425383 PMC7028471

[ref33] Myćka G. Ropka-Molik K. Cywińska A. Szmatoła T. Stefaniuk‐Szmukier M. (2023). Molecular insights into the lipid-carbohydrates metabolism switch under the endurance effort in Arabian horses. Equine Vet. J. 56, 586–597. doi: 10.1111/evj.13984, PMID: 37565649

[ref34] Newell M. L. Wallis G. A. Hunter A. M. Newell M. Wallis G. Hunter A. . (2018). Metabolic responses to carbohydrate ingestion during exercise: associations between carbohydrate dose and endurance performance. Nutrients 10:37. doi: 10.3390/nu10010037, PMID: 29301367 PMC5793265

[ref35] Nieman D. C. Sakaguchi C. A. Williams J. C. Lawson J. Lambirth K. C. Omar A. M. . (2025). Gut *Prevotella copri* abundance linked to elevated post-exercise inflammation. J. Sport Health Sci. 14:101039. doi: 10.1016/j.jshs.2025.101039, PMID: 40194740 PMC12145743

[ref36] Ochten N. A. V. Suckow E. Forbes L. . (2025). The structural and functional aspects of exercise-induced cardiac remodeling and the impact of exercise on cardiovascular outcomes. Ann. Med. 57:2499959. doi: 10.20022/j.cnki.1000-1166.2022050003 40377449 PMC12086911

[ref37] Ouyang W. Qi J. Z. Si R. B. Wang D. Zheng W. X. Wang W. . (2023). Key points of feeding and management for young sportive Yili horses. Xinjiang Animal Husbandry 39, 26–30. doi: 10.16795/j.cnki.xjxmy.2023.4.004

[ref38] Peng X. Wang t. Meng j. . (2024). Correlation analysis of cardiac dimensions and racing performance in 2 years old Yili horses. J. Vet. Med. 55, 2963–2972. Available at: https://kns.cnki.net/KCMS/detail/detail.aspx?dbcode=CJFQ&dbname=CJFDLAST2024&filename=XMSY202407016

[ref39] Pınar O. Sancak A. A. (2018). Effects of different heart dimensions on race performance in thoroug bred race horses. Acta Sci. Vet. 46:7. doi: 10.22456/1679-9216.84209

[ref40] Qiu P. Ishimoto T. Fu L. Zhang J. Zhang Z. Liu Y. (2022). The gut microbiota in inflammatory bowel disease. Front. Cell. Infect. Microbiol. 12:3992. doi: 10.3389/fcimb.2022.733992, PMID: 35273921 PMC8902753

[ref42] Santos M. M. Ramos G. V. de Figueiredo I. M. Silva T. C. B. V. Lacerda-Neto J. C. (2023). Cardiac changes after lactate-guided conditioning in young purebred Arabian horses. Animals 13:1800. doi: 10.3390/ani13111800, PMID: 37889733 PMC10252023

[ref43] Solouki S. Gorgani-Firuzjaee S. Jafary H. Delfan M. (2024). Efficacy of high-intensity interval and continuous endurance trainings on cecal microbiota metabolites and inflammatory factors in diabetic rats induced by high-fat diet. PLoS One 19:e0301532. doi: 10.1371/journal.pone.0301532, PMID: 38626052 PMC11020751

[ref44] Tanase D. M. Gosav E. M. Neculae E. Costea C. F. Ciocoiu M. Hurjui L. L. . (2020). Role of gut microbiota on onset and progression of microvascular complications of type 2 diabetes (T2DM). Nutrients 12:3719. doi: 10.3390/nu12123719, PMID: 33276482 PMC7760723

[ref45] Tanca A. Palomba A. Fiorito G. Abbondio M. Pagnozzi D. Uzzau S. (2024). Metaproteomic portrait of the healthy human gut microbiota. Npj Biofilms Microb 10:54. doi: 10.1038/s41522-024-00526-4, PMID: 38944645 PMC11214629

[ref46] Tao J.-s. Su S.-f. Zhang J.-q. Wu H. Q. Zhao J. L. Hu H. (2021). Research advances on microbial composition in horse intestinal tract and influencing factors. Anim. Husbandry Feed Sci. 42, 61–66. Available at: https://kns.cnki.net/KCMS/detail/detail.aspx?dbcode=CJFQ&dbname=CJFDLAST2021&filename=NMXK202104012

[ref47] Vasco C. Brinkley-Bissinger K. Paschoal V. R. Lance J. (2020). 110 fecal pH, dry matter and volatile fatty acids of horses grazing legume-grass mixed pastures. J. Anim. Sci. 98, 90–90. doi: 10.1093/jas/skaa278.164, PMID: 32704858

[ref48] Wang T. Meng J. Wang J. Ren W. Yang X. Adina W. . (2025). Absolute quantitative Lipidomics reveals differences in lipid compounds in the blood of trained and untrained Yili horses. Vet. Sci. 12:255. doi: 10.3390/vetsci12030255, PMID: 40266993 PMC11945474

[ref49] Wang J. Zhu G. Sun C. Xiong K. Yao T. Su Y. . (2020). TAK-242 ameliorates DSS-induced colitis by regulating the gut microbiota and the JAK2/STAT3 signaling pathway. Microb. Cell Factories 19:158. doi: 10.1186/s12934-020-01417-x, PMID: 32762699 PMC7412642

[ref50] Witkowska-Piłaszewicz O. Pingwara R. Winnicka A. (2020). The effect of physical training on peripheral blood mononuclear cell ex vivo proliferation, differentiation, activity, and reactive oxygen species production in racehorses. Antioxidants 9:1155. doi: 10.3390/antiox9111155, PMID: 33233549 PMC7699811

[ref51] Wu N. Li X. Ma H. Zhang X. Liu B. Wang Y. . (2023). The role of the gut microbiota and fecal microbiota transplantation in neuroimmune diseases. Front. Neurol. 14:8738. doi: 10.3389/fneur.2023.1108738, PMID: 36816570 PMC9929158

[ref52] Wu Q. Zhang H.-r. Sun J.-d. Chen Y. B. Tian X. Lan H. L. . (2024). Evaluation of biventricular myocardial strain in amateur marathoners by cardiovascular magnetic resonance imaging with feature tracking technique. Radiol Pract 39, 1051–1058. doi: 10.13609/j.cnki.1000-0313.2024.08.011

[ref53] Xia W. Li X. Han R. Liu X. (2024). Microbial champions: the influence of gut microbiota on athletic performance via the gut-brain Axis. Open Access J. Sports Med. 15, 209–228. doi: 10.2147/OAJSM.S485703, PMID: 39691802 PMC11651067

[ref41] Yang W. Xu Y. Wu P. Chen J. Cai Y. Zhou J. (2019). Characteristics of the gut microbiota in professional martial arts athletes: A comparison between different competition levels. Plos One. 14:e0226240. doi: 10.1371/journal.pone.0226240 31881037 PMC6934331

[ref54] Yang J. Zhang W. Dong C. (2025). Gut microbiota alteration with moderate-to-vigorous-intensity exercise in middle school female football athletes. Biology 14:211. doi: 10.3390/biology14020211, PMID: 40001979 PMC11852635

[ref55] Yokoyama S. Hayashi M. Goto T. Muto Y. Tanaka K. (2023). Identification of cfxA gene variants and susceptibility patterns in β-lactamase-producing Prevotella strains. Anaerobe 79:102688. doi: 10.1016/j.anaerobe.2022.102688, PMID: 36580990

[ref56] Yu C. Liu S. Chen L. Shen J. Niu Y. Wang T. . (2019). Effect of exercise and butyrate supplementation on microbiota composition and lipid metabolism. J. Endocrinol. 243, 125–135. doi: 10.1530/JOE-19-0122, PMID: 31454784

[ref57] Zhao Y. C. Gao B. h. (2024). Integrative effects of resistance training and endurance training on mitochondrial remodeling in skeletal muscle. Eur. J. Appl. Physiol. 124, 2851–2865. doi: 10.1007/s00421-024-05549-5, PMID: 38981937

