# Supplementary material for: Gut microbial signatures and cardiac-microbiota axis in Yili horses with divergent exercise-induced cardiac remodeling
Source: Front Microbiol. 2025 Dec 3;16:1689293. doi: 10.3389/fmicb.2025.1689293 (PMC12711142; doi:10.3389/fmicb.2025.1689293)
Supplement: Supplementary file 1 [file Data_Sheet_1.zip › Supplementary Table/Table 1.docx]

| **Sample** | **CleanData_bases/G** | **CleanData_Q20/%** | **CleanData_Q30/%** | **CleanData_GC/%** |
| --- | --- | --- | --- | --- |
| D.1 | 13.15 | 97.98 | 95.66 | 45.58 |
| D.2 | 10.88 | 97.89 | 95.44 | 44.91 |
| D.3 | 12.2 | 97.89 | 95.44 | 44.92 |
| D.4 | 12.16 | 97.91 | 95.53 | 44.33 |
| D.5 | 9.35 | 98.1 | 95.84 | 45.77 |
| D.6 | 14.3 | 97.91 | 95.53 | 42.81 |
| D.7 | 13.32 | 98.03 | 95.78 | 44.08 |
| D.8 | 12.54 | 98 | 95.63 | 45.12 |
| D.9 | 13.46 | 98.26 | 96.16 | 46.11 |
| D.10 | 11.47 | 98.31 | 96.28 | 44.06 |
| D.11 | 13.56 | 97.97 | 95.65 | 43.73 |
| D.14 | 11.76 | 99.14 | 97.73 | 46.88 |
| D.15 | 11.18 | 99.06 | 97.55 | 45.85 |
| D.16 | 11.76 | 99 | 97.41 | 47.14 |
| D.18 | 11.57 | 99.11 | 97.66 | 46.71 |
| D.20 | 11.15 | 98.96 | 97.3 | 47.45 |
| D.22 | 9.98 | 99.01 | 97.42 | 46.88 |
| D.23 | 11 | 99.11 | 97.62 | 46.58 |
| D.24 | 10.78 | 99.03 | 97.42 | 47.12 |
| D.25 | 10.51 | 98.98 | 97.35 | 49.05 |
